# Humanistic nursing care management strategies: from formulation to implementation

**DOI:** 10.3389/fpubh.2025.1591077

**Published:** 2025-05-12

**Authors:** Jing Lv, Yajie Su, Hongmei Tang, Xiaolin Jiang, Xiaojuan Chen

**Affiliations:** ^1^Department of Orthopedics, Ziyang Central Hospital, Ziyang, China; ^2^Department of Gastroenterology, Ziyang Central Hospital, Ziyang, China; ^3^Nursing Department, Ziyang Central Hospital, Ziyang, China

**Keywords:** humanistic care, nursing management, nurses, inpatient, caring model

## Abstract

**Objective:**

To investigate the clinical implementation and effectiveness of the humanistic care nursing model, and to offer a reference for enhancing humanistic care practices in clinical settings.

**Methods:**

Three hundred and eighteen nurses and five hundred and two inpatients were selected as the control group and observation group before and after the implementation of humanistic care model in China from October to November 2023 and from May to June 2024. The differences of humanistic care ability, humanistic care behavior, medical narrative ability, nurses’ perception of the hospital’s attention to their caring ability, patients’ and nurses’ satisfaction, and patients’ evaluation of nurses’ caring behavior were compared between the two groups.

**Results:**

After the implementation of the humanistic care model, the total score of nurses’ humanistic care ability, humanistic care behavior, medical narrative ability and scores of all dimensions were significantly increased, nurses’ perception of the hospital’s attention to their caring ability was significantly increased, patients’ evaluation of nurses’ caring behavior was improved, and both patients’ and nurses’ satisfaction were increased, with statistical significance (*p* < 0.05).

**Conclusion:**

The implementation of humanistic care nursing model can effectively improve managers’ care for nurses, further improve nurses’ humanistic care ability, humanistic care behavior and medical narrative ability, patients have a higher evaluation of nurses’ caring behavior, and nurses’ and patients’ satisfaction has been improved.

**Implications for nursing management:**

We recommend that hospital administrators not only provide care for patients but also extend their support to nurses. It is essential to continuously optimize the “care chain” among nurse managers, nurses, and patients.

## 1 Introduction

The transformation of medical models has enriched the connotation of nursing work, driving nursing services toward a more humanized and high-quality development. Care is the core of nursing (1). It not only manifests as specific behaviors but also embodies profound compassion and attitude (2). Positive caring behaviors can effectively promote patient recovery (3), enhance patient satisfaction (4), and improve the quality of nursing care (5). The “National Nursing Development Plan (2021–2025)” explicitly emphasizes the importance of strengthening humanistic care in nursing and implementing a comprehensive responsibility system for holistic nursing (6). The Watson Caring Theory overly relies on the individual moral values of nurses, neglecting the role of organizational factors in shaping caring behaviors. Currently, there exists a contradiction in nursing practice between the excessive emotional labor required from nurses and the escalating humanistic needs of patients (7). The assessment system within medical institutions remains predominantly focused on technical indicators, lacking quantitative evaluation standards for humanistic care (6). A survey conducted across 188 medical institutions regarding the current state of humanistic nursing practices indicates that such practices are generally at a moderately high level; however, they exhibit fragmented characteristics (8).

As the concept of humanistic care becomes increasingly ingrained, researchers have developed numerous initiatives based on clinical practice to explore fundamental models of humanistic care. These efforts have yielded positive outcomes in enhancing patients’ healthcare experiences and improving treatment results (9). Dingman et al. (10), drawing on Watson’s Theory of Caring, has developed a patient care model centered on five core caring behaviors. These behaviors encompass: introducing oneself to the patient and clearly elucidating one’s role; addressing the patient by their preferred form of address; dedicating at least 5 min at the patient’s bedside during each shift change to review and develop the care plan; conveying care through non-verbal gestures such as handshakes or gentle touches on the arm; and incorporating the organization’s mission, vision, and values into the formulation of nursing plans. The overarching goal of this model is to enhance patient satisfaction. Lukose (11) has developed a humanistic care practice model based on Watson’s theory of caring. This model emphasizes the interaction between nurses and patients, guided by 10 essential elements of care and caregiving procedures. By establishing interpersonal caring relationships, authentic connections, and moments or scenarios of care between nurses and patients, it fosters a healing environment through tolerance, compassion, and love to achieve patient-centered care. The aforementioned model exhibits a dual flaw characterized by an emphasis on “patients over nurses” and “concepts over systems. In our country, research on the theory of humanistic care is relatively limited. Many researchers base their studies on an understanding of the fundamental connotations of humanistic care and related foreign theories. The lack of a structured and scientific guiding model in our nation hinders the implementation of humanistic care practices, significantly restricting the effectiveness of these initiatives. Therefore, it is particularly important to actively explore a scientifically sound and effective model. This study proposes a dual-subject model of humanistic care that is “employee-centered and patient-oriented.” By inheriting Watson’s core theory of person-centeredness and applying psychological contract theory (12), it establishes an emotional labor protection framework for nurses. This approach transforms humanistic care from ethical advocacy into institutional practice, thereby addressing the complex demands of the healthcare landscape in China. This model not only addresses the limitations of existing theories, which often “prioritize patients over nurses” and “favor concepts over systems,” but also substantiates the significance of dual-subject synergy in enhancing the quality of nursing. Based on this premise, the present study aims to investigate the effects of this model on nurses’ humanistic care abilities, humanistic care behaviors, medical narrative skills, and overall satisfaction. Additionally, it will explore whether patients’ evaluations of nurses’ care behaviors and their satisfaction levels have improved as a result.

## 2 Participants and procedure

Three hundred and eighteen nurses and five hundred and two inpatients were selected as the control group and observation group before and after the implementation of humanistic care model in China from October to November 2023 and from May to June 2024. Inclusion criteria for nurses: Possession of a valid nursing license and registration, informed consent, and voluntary participation in this study. Exclusion criteria for nurses: Those who are on maternity leave or absent from work for other reasons during the survey period, interns and trainees, nurses who withdraw midway due to special circumstances, as well as those engaged in nursing management or teaching and research activities. Inclusion criteria for patients: Patients must have stable conditions without any mental disorders, a hospitalization duration of at least 3 days, voluntarily agree to participate in the survey, and be capable of independently completing the questionnaire. Exclusion criteria for hospitalized patients: Patients with mental or cognitive impairments who are unable to cooperate in filling out the questionnaire, as well as those who are being readmitted for a second time.

The minimum sample size was determined by estimating the sample size according to the WHO recommendations for epidemiological studies (13). The confidence interval (CI) was set at 95%, the standard deviation (SD) was 0.5, and the margin of error was 0.5. Additionally, a 10% contingency was added to account for non-response. The nursing group conducted cluster sampling based on the nursing unit. The researcher distributed questionnaires during the daily morning meetings and collected them on-site. For the patient group, systematic sampling was performed based on admission time, followed by bedside surveys conducted by trained research assistants between 15:00 and 17:00 each day. Initially, 318 nurses were included in the study, however, 18 were excluded for various reasons: four due to job changes and 14 for not completing the post-intervention questionnaire. A total of 502 patients were initially enrolled, but 86 were excluded, this included 62 who could not complete follow-up after intervention and 24 identified as outliers in the data. This study was reviewed and approved by the Ethics Committee of our hospital (No. 2023-380). Also, written consent was received from the participants to participate in the study. A comparison of general data between the two groups of nurses and hospitalized patients revealed no statistically significant differences (*p* > 0.05) (Tables 1, 2).

**Table 1:** Comparison of general data between the two groups of nurses.

Items		Before implementation (*n* = 318)	After implementation (*n* = 318)	χ^2^/*t*	*P*
Sex	Male	12 (3.8)	11 (3.5)	0.045	0.832
	Female	306 (96.2)	307 (96.5)		
Age (years)	18–30	156 (49.1)	149 (46.9)	−0.339	0.735
	31–40	148 (46.5)	157 (49.4)		
	41–50	12 (3.8)	10 (3.1)		
	51–60	2 (0.6)	2 (0.6)		
Working years	≥21	14 (4.4)	10 (3.1)	1.748	0.626
	11–20	92 (28.9)	84 (26.4)		
	6–10	100 (31.4)	99 (31.1)		
	≤5	112 (35.2)	125 (39.3)		
Education	Bachelor degree or above	270 (84.9)	261 (82.1)	4.4	0.111
	College or vocational	48 (15.1)	53 (16.7)		
	Technical secondary school	0 (0.0)	4 (1.2)		
Marital status	Unmarried	84 (26.4)	69 (21.7)	2.492	0.477
	Married	224 (70.4)	241 (75.8)		
	Divorced	8 (2.5)	7 (2.2)		
	Widowed spouse	2 (0.6)	1 (0.3)		
Positional titles	Nurse	46 (14.5)	34 (10.7)	2.708	0.439
	Nurse practitioner	134 (42.1)	135 (42.5)		
	Supervisor nurse	130 (40.9)	143 (45)		
	Associate senior nurse and above	8 (2.5)	6 (1.9)		

**Table 2:** Comparison of general data between the two groups of hospitalized patients.

Items		Before implementation (*n* = 502)	After implementation (*n* = 502)	χ^2^/*t*	*P*
Sex	Male	206 (41)	220 (43.8)	0.799	0.371
	Female	296 (59)	282 (56.2)		
Age (years)	18 ~ 40	186 (37.1)	208 (41.4)	2.772	0.25
	41 ~ 65	250 (49.8)	224 (44.6)		
	>65	66 (13.1)	70 (13.9)		
Education	Bachelor degree or above	126 (25.1)	118 (23.5)	2.212	0.53
	College or vocational	94 (18.7)	110 (21.9)		
	High school and secondary school	78 (15.5)	68 (13.5)		
	Junior high and below	204 (40.6)	206 (41)		
Marriage	Unmarried	104 (20.7)	136 (27.1)	5.692	0.128
	Married	358 (71.3)	330 (65.7)		
	Divorced	10 (2)	10 (2)		
	Widowed spouse	30 (6)	26 (5.2)		
Place of residence	City	278 (55.4)	252 (50.2)	2.987	0.225
	Township	86 (17.1)	102 (20.3)		
	Rural	138 (27.5)	148 (29.5)		

## 3 Methods

### 3.1 Establishment of a humanistic care management team

The group is led by the Nursing Department, with the director as the group leader and 2 head nurses as the deputy group leaders. The group members include 2 associate senior nurse, 2 national second-level psychological counselors and 3 nursing graduate students. The group leader is responsible for the overall arrangement of humanistic activities, and the deputy group leader is responsible for the overall implementation plan and quality control. The rest of the team is responsible for searching relevant literature at home and abroad, constructing humanistic nursing model, training, on-site hosting, completing investigation tasks and commenting on the training.

### 3.2 The construction of a humanistic care nursing model

The team leader will articulate the objectives and significance of the research to the directors of participating departments and head nurses. Additionally, a comprehensive one-week training program will be conducted for members of the humanistic care group. Implementation will only proceed once all group members have successfully passed their assessments.

**Figure 1:**
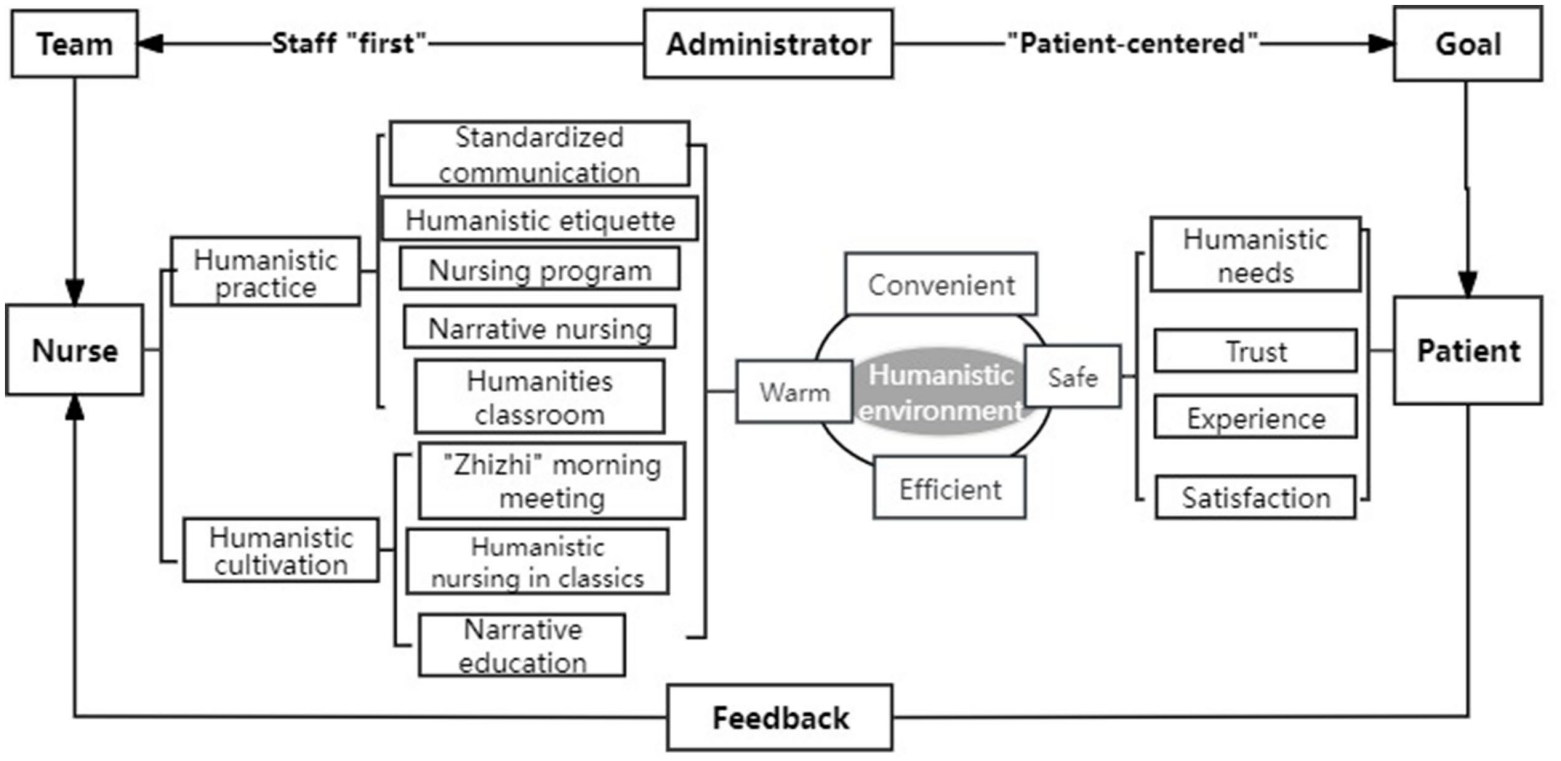
Structure diagram of humanistic care nursing model.

We adopt the project management system, bring together nursing elites, and build a humanistic care nursing model of staff “first” and “patient-centered” (Figure 1). First of all, adhere to the staff “first” as the basis, protect the rights and interests of employees, formulate training plans, strengthen the humanistic care awareness of nurses, improve humanistic quality and humanistic care practice ability. At the same time, we are committed to the “patient-centered” principle, to create a safe, convenient, efficient and warm medical environment, to provide patients with comprehensive and multi-dimensional care behavior.

### 3.3 Cultivation of humanistic quality

#### 3.3.1 Innovate humanities classroom and improve humanistic literacy

This training course is grounded in the theory of humanistic care (14) and integrates transformational learning theory (15), social cognitive theory (16), as well as the cultivation and practice of humanistic literacy (17, 18). The objective is to construct a comprehensive training system characterized by theoretical immersion—behavioral transformation—practical integration. Firstly, within the framework of humanistic care theory, an in-depth analysis of Watson’s elements of caring is conducted. For instance, a dignity maintenance workshop specifically designed for chemotherapy patients has been established. This workshop simulates the psychological needs associated with hair loss and requires nurses to employ language that affirms feelings while documenting patients’ emotional changes. Drawing on social cognitive theory, communication skills training has been developed to progressively enhance nurses’ abilities to manage conflicts across varying scenarios—from low-stakes situations to high-conflict environments. Furthermore, the cultivation and practice of humanistic literacy serve as a means to assess nurses’ competencies in providing humanistic care. This ensures that they can adeptly apply their acquired knowledge in real-world settings. For example, role-playing activities are implemented to simulate patient care under diverse circumstances—such as interacting with anxious patients or emotionally unstable family members—thereby enabling nurses to learn how to deliver more compassionate care services through practical experience.

#### 3.3.2 Nurses experience caring and improve the working atmosphere

(1) Conducting various activities such as Balint groups and team-building exercises, these sessions facilitate collective interaction and discussion. we aim to assist nurses in addressing psychological issues, enhancing self-awareness, and improving social skills, thereby fostering the overall competence and teamwork abilities of nursing professionals. (2) In the context of unchanged hospital hardware facilities, the implementation of a self-scheduling app for nurses will optimize the allocation of nursing resources and better meet the needs of nursing staff. (3) Establish a Nurse Sleep Day, during which nurses are exempt from attending departmental and hospital meetings as well as training sessions. They will also not be required to undertake secondary duties on this day. (4) The nursing staff in the ward evaluates the head nurse based on four criteria: “morality,” “capability,” “diligence,” and “performance.” Through the analysis and feedback of the assessment forms, this process aims to enhance the management skills of the head nurse. (5) Online learning and remote guidance have transcended geographical and temporal limitations, providing nurses with greater flexibility and autonomy. (6) To establish a reading corner for nurses, providing them with a quiet and comfortable environment amidst their busy work schedule. This space allows them to relax and enjoy the pleasure of reading, featuring a diverse collection of books covering various fields such as medicine, nursing, psychology, and humanities and social sciences.

#### 3.3.3 Implement the “Zhizhi” morning meeting to improve humanistic literacy

Through daily “Zhizhi” morning meetings for collective learning and exchange, we aim to enhance nurses’ cultural literacy and overall competence. The content includes sharing of humanistic knowledge, recitation of classics, exchange of insights, and discussion of various topics. We encourage nurses to learn in a relaxed and enjoyable atmosphere, share their work experiences, and listen to the stories of others. This process allows them to draw wisdom and inspiration while cultivating their empathy and compassion skills.

#### 3.3.4 Studying the humanistic care found in classical texts awakens our original commitment to humanity

Through in-depth study and analysis of ancient literature, the concept of “Great Physician with Sincere Heart” is applied to the cultivation of humanistic qualities in hospitals. In conjunction with contemporary nursing practices, we implement a regular weekly learning program that includes article dissemination. This initiative aims to help nursing staff deeply appreciate the value and significance of the “Great Physician with Sincere Heart” philosophy within their caregiving roles, thereby enhancing nurses’ understanding and recognition of ancient humanistic care principles. Gradually, this understanding will be internalized as part of their professional values, promoting a parallel development between practical skills assessment and ethical evaluation in their profession.

#### 3.3.5 Implement narrative education to enhance empathy skills

In the training sessions, each class will incorporate 5–10 min of narrative segments. These segments will include selected classic care stories and videos from both domestic and international sources. Through the recitation of narrative cases and role-playing scenarios, we aim to assist nurses in better understanding the feelings and experiences of others. This approach highlights the compassion and support that nurses provide to patients during critical moments, allowing them to intuitively grasp the real-life context of nursing work. It is designed to engage nurses’ interest and participation while enabling them to experience the warmth and value of nursing, ultimately enhancing their empathetic abilities.

### 3.4 Humanistic practice

#### 3.4.1 Three standardized communication models to improve humanistic communication skills in different scenarios

To ensure the accurate transmission of information and facilitate efficient communication, we have developed standardized procedures tailored to various scenarios. These procedures are grounded in the six key steps of the CICARE communication model (19), the five fundamental elements of the AIDET communication model (20), and the four core components of the SBAR communication model (21). The CICARE model emphasizes building trust between nurses and patients while enhancing empathy through standardized behaviors and open-ended questions. This model is particularly applicable to daily education initiatives and health promotion activities. Conversely, the AIDET model focuses on managing patient anxiety by reducing uncertainty through transparent information sharing and emotion-naming techniques; it is predominantly utilized in high-stress situations such as examination processes and disease notifications. The SBAR model plays a crucial role in ensuring both accuracy and timeliness in cross-professional information exchange. It necessitates structured statements to achieve zero-error teamwork, making it primarily relevant for shift handovers and critical incident reporting. Collectively, these models enhance nurse–patient communication efficiency while simultaneously decreasing nursing complaints by progressing from relationship-building to anxiety alleviation, ultimately leading to effective information integration.

#### 3.4.2 Standardize humanistic nursing etiquette, fully respecting and caring for patients

We invited airline trainers to conduct etiquette training for nursing staff. The training content includes sincere attitudes, humble and elegant language, and the appropriate choice of words in communication; professional appearance, beautiful language, graceful interaction, and refined behavior; as well as grooming etiquette before, during, and after procedures—encompassing verbal communication, body language, and overall conduct. Experts provided on-site guidance for nurses in practicing humanistic etiquette. This not only enhanced the professional image of the nursing staff but also strengthened patients’ trust in the medical team and increased their satisfaction levels.

#### 3.4.3 List management enhances nursing procedures, strengthening the foundation for safety

The principle of “patient-centered care” is upheld, drawing on the scientific problem-solving approaches and methods inherent in nursing procedures. By integrating the characteristics of humanistic nursing, we employ the primary steps of the nursing process—namely “assessment, planning, implementation, and evaluation” (22), to develop a scientifically sound and standardized nursing checklist. This checklist encompasses admission assessment, preoperative verification, postoperative education, postoperative care, and discharge preparation lists. It aligns with the individualized needs of patients to ensure they receive timely and effective compassionate services.

#### 3.4.4 Utilizing narrative practices to enhance nurses’ empathy

Each department has established a narrative nursing group, implementing scientific and effective care measures for patients in accordance with the phased requirements of attention, understanding, reflection, and response (23). The “Parallel Nursing Records” (24) are utilized to objectively and authentically describe the challenges, difficulties, and suffering faced by patients and their families during the medical process. Communication and learning occur through two formats: sharing narrative stories and conducting narrative nursing rounds. This practice enhances nurses’ ability to apply narrative nursing techniques effectively. Regularly featured in our hospital’s official bulletin board under the “Nursing Listening” column are touching stories shared with patients that convey compassion and hope. Outstanding cases of narrative nursing will be evaluated, recognizing individuals and teams who excel in this field to inspire more nurses to actively participate.

### 3.5 Create a humanistic environment in the hospital

We actively respond to the needs of specialized departments by creating a “One Specialty, One Feature” humanistic ward. This includes the development of exhibition boards for humanistic care services and a wish wall, prominently displayed in the department to intuitively convey our philosophy of humanistic care and establish new communication bridges among patients, nurses, and family members. In terms of ward decoration, we utilize warm color schemes and user-friendly designs to create a comfortable and tranquil medical environment. The oncology department has adopted the “Light of Life” theme, implementing a color temperature adaptive lighting system designed to minimize the frequency with which patients awaken during the night. Meanwhile, the pediatric ward has established a “Forest Wonderland” ecosystem that utilizes 3D dynamic projections to help alleviate preoperative anxiety in children. For nursing services, we provide convenient facilities such as shared wheelchairs and convenience boxes (containing tissues, cups, umbrellas, hairdryers, sewing kits, adhesive bandages), as well as drinking water dispensers. Regarding dietary provisions, we offer nutritionally balanced meals with diverse flavors to meet patient needs. Our aim is to deliver efficient, convenient, safe, and warm medical services while fostering a strong humanistic atmosphere that contributes to both a positive departmental environment and an effective working setting.

### 3.6 Effectiveness evaluation

#### 3.6.1 Caring ability inventory

It was a scale developed by Nkongho (25) to measure the humanistic care abilities of university students from various disciplines, including nursing and pharmacy. This measurement tool encompasses three dimensions: cognition, courage, and patience, comprising a total of 37 items. The Cronbach’s α coefficient for this scale is 0.840, indicating it is one of the most widely used scales both domestically and internationally. A revised Chinese version of the scale was adapted and translated by Xu et al. (26), specifically for assessing the humanistic care abilities of hospital nurses, also consisting of 37 items across three dimensions. The scale employs a 7-point Likert scoring system, ranging from “strongly disagree” to “strongly agree,” with scores assigned from 1 to 7. Notably, item 13 is scored in reverse. The total score can range from 37 to 259 points, with higher scores indicating stronger humanistic caring abilities. The Cronbach’s α coefficient for the scale is reported at 0.84, while the content validity stands at 0.78. A total score exceeding 210.53 indicates high caring ability; scores between 171.55 and 210.53 reflect moderate caring ability; and scores below 171.55 signify low caring ability (27).

#### 3.6.2 Caring behaviors inventory

The questionnaire developed by Wolf et al. (28), is designed to measure the caring abilities of patients and nurses. The measurement dimensions include: respect for individual differences, attention to others’ experiences, ensuring humanity, positive engagement, professional knowledge and skills. It consists of 42 items and employs a Likert four-point rating scale, with a Cronbach’s α coefficient of 0.830. The scale has been adapted into Chinese by Da et al. (29), allowing for evaluations both from patients regarding nurses’ caring behaviors and self-assessments by nurses themselves. This version encompasses three dimensions: respect and connection, knowledge and skills, as well as support and assurance, comprising a total of 24 items. It employs a six-point Likert scale, where scores range from 1 (“Never”) to 6 (“Always”). A higher total score indicates a greater level of care provided by nurses to patients. The Cronbach’s α coefficient for this scale is 0.959.

#### 3.6.3 Narrative competence scale

It was developed by Ma et al. (30), to evaluate the narrative competence of healthcare professionals. It encompasses three dimensions: attentive listening, understanding responses, and reflective representation, comprising a total of 27 items. Each item is rated using a 7-point Likert scale, with an overall score ranging from 27 to 189 points; higher scores indicate greater medical narrative competence. The Cronbach’s α coefficient for this scale is reported to be 0.950.

#### 3.6.4 The patient satisfaction with nursing care

Liu et al. (31) developed a questionnaire in 2014, which consists of 7 dimensions and 26 items. Each item employs a Likert 5-point scale ranging from “very satisfied” to “dissatisfied,” with corresponding scores assigned from 5 to 1. A higher score indicates greater satisfaction. The Cronbach’s α coefficient for the questionnaire is 0.78, and the Content Validity Index (CVI) for each item exceeds 0.81.

#### 3.6.5 Caring behavior evaluation scale

The questionnaire revised by Liu et al. (31), is intended for the evaluation of nurses’ caring behaviors from the perspective of hospitalized patients. It consists of 22 items and employs a 5-point Likert scale ranging from “Always” to “Never.” Each item is scored from 5 to 1, with higher total scores indicating greater nursing care ability. The Cronbach’s α coefficient for this questionnaire is 0.831, and the test–retest reliability is measured at 0.86.

### 3.7 Statistical methods

The data were entered and analyzed of SPSS 26.0. For normally distributed continuous variables, the results were presented as mean ± standard deviation (*x− ± s*) and analyzed using *t*-tests. Categorical data were expressed in terms of frequency and percentage (%), with chi-square tests employed for analysis. The significance level was set at α = 0.05, with *p* < 0.05 indicating statistical significance in differences observed.

## 4 Results

The results of this study indicate that after the implementation of the humanistic care nursing model, overall scores for nurses’ abilities in humanistic care, medical narrative skills, and humanistic care behaviors, as well as scores across various dimensions were significantly higher than those prior to implementation (*p* < 0.05) (Table 3). Furthermore, nurses’ perceptions of the hospital’s emphasis on their well-being and their job satisfaction markedly improved compared to before implementation (*p* < 0.01) (Table 4). Additionally, patients’ evaluations of nurses’ caring behaviors and patient satisfaction showed a significant increase relative to pre-implementation levels, also demonstrating statistical significance (*p* < 0.01) (Table 5).

**Table 3:** Comparison of humanistic care ability, medical narrative ability and humanistic care behavior of nurses between the two groups (score, *x− ± s*) (*n* = 300).

Items	Before implementation	After implementation	*T*-value	*P*
Human caring ability	204.19 ± 22.08	215.14 ± 12.76	−5.16	0.00
Cognition	86.17 ± 10.32	89.93 ± 7.11	−3.68	0.00
Courage	54.58 ± 13.81	59.29 ± 7.05	−3.65	0.00
Patience	62.75 ± 6.11	65.92 ± 4.68	−5.00	0.00
Medical narrative ability	167.99 ± 21.67	177.29 ± 11.49	−4.81	0.00
Attentive listening	54.83 ± 7.30	59.42 ± 4.13	−6.85	0.00
Understanding Responses	75.28 ± 10.24	78.77 ± 5.29	−3.82	0.00
Reflective reproduction	37.89 ± 5.06	39.09 ± 3.02	−2.63	0.009
Humanistic care behavior	131.65 ± 16.44	136.83 ± 8.69	−3.47	0.001
Respect and connection	53.54 ± 8.31	56.60 ± 4.15	−4.08	0.000
Knowledge and skills	27.84 ± 3.24	28.77 ± 1.83	−3.18	0.002
Support and assurance	50.27 ± 5.76	51.47 ± 3.67	−2.14	0.034

**Table 4:** Comparison of nurses’ perceptions of hospital care and attention levels with nurse satisfaction (score, *x− ± s*) (*n* = 300).

Items	Before implementation	After implementation	*T*-value	*P*
Nurses perceive the care for them from the hospital	1.23 ± 0.47	3.78 ± 0.46	−47.32	0.00
Nurse satisfaction	81.92 ± 15.54	95.11 ± 9.53	15.45	0.000

**Table 5:** Evaluation of patient satisfaction and perception of nursing care behaviors (score, *x− ± s*) (*n* = 416).

Items	Before Implementation	After implementation	*T*-value	*P*
Patients’ evaluation of nurses’ caring behavior	81.99 ± 15.35	107.55 ± 6.19	−21.80	0.000
Patient satisfaction	95.34 ± 11.39	120.74 ± 7.84	−27.40	0.000

## 5 Discussion

### 5.1 The importance of establishing a humanistic care nursing model

As research on humanistic care in nursing continues to deepen, scholars both domestically and internationally are actively exploring various specific measures and actions for implementing humanistic care in clinical practice. However, a clear model of humanistic care remains lacking. The humanistic care model serves as a crucial bridge that transforms the concept of humanistic care from theory into practice. In the process of advancing the implementation of humanistic care in nursing, it comprehensively integrates foundational theories and core elements of humanistic care, constructs standardized processes and measures for providing such care, and establishes a model grounded in theoretical principles while being oriented toward clinical practice. This aims to promote the evolution of nursing practices related to humanistic care toward greater scientific rigor, standardization, and normalization. Ultimately, this ensures the philosophy of humanistic care is thoroughly, deeply, and effectively integrated into nursing practices (9).

The subjects of humanistic care primarily encompass the concern that nurses have for patients, as well as the attention that nursing managers extend to their staff (32). Based on this understanding, we have established an employee-centered humanistic care model that prioritizes patient needs. This model delineates specific and practical measures and behaviors related to humanistic care within clinical practice, while continuously optimizing the “care chain” among nurse managers, nurses, and patients. Through the transmission and diffusion of love, along with the guidance and promotion of care initiatives, we aim to foster a positive interaction and cyclical relationship. In practicing humanistic care, we not only deepen emotional connections among medical staff, nurses, and patients but also further reinforce nursing personnel’s sense of professional mission. This encourages them to engage more proactively in their work, thereby enhancing both work efficiency and overall quality of nursing services. Ultimately, this approach allows us to provide superior and more humane care for our patients (33).

### 5.2 The implementation of the humanistic care nursing model can effectively enhance nurses’ abilities in humanistic care, medical narrative skills, and behaviors related to humanistic care

The current emphasis on humanistic care capabilities has become a core element in assessing the comprehensive quality of clinical nurses (34). It necessitates that nurses actively engage emotionally in their work while possessing solid skills in humanistic care. Research indicates that, at present, nurses exhibit relatively weak abilities in this area, with limited demonstration of humanistic care within nursing practice. Therefore, there is an urgent need for targeted training programs to enhance nurses’ competencies in humanistic care (35). This study organized various activities for nurses, including innovative theoretical learning sessions on humanism, “Zhizhi” morning meetings focused on sharing knowledge, narrative education, recitation of classics, sharing insights, and topic discussions. The aim was to elevate their professional knowledge and cultural literacy so that they could deeply understand the core concepts of humanistic care and apply them effectively in clinical settings. Results showed that the scores reflecting nurses’ capabilities in humanistic care increased from (204.19 ± 22.08) before implementation to (215.14 ± 12.76) after implementation (*p* < 0.001), consistent with findings by Hu et al. (36). Through systematic learning of relevant knowledge related to humanity, nurses deepened their understanding and internalization of the essence of nursing’s humanistic care principles, resulting in improved cognitive awareness (*p* < 0.01). By engaging in self-directed learning and reflective writing through narrative diaries documenting patients’ psychological changes during illness experiences, they gained deeper insight into the suffering associated with disease as well as issues surrounding life and death, thereby enhancing their patience (*p* < 0.01). Furthermore, based on improvements in patient cognition and patience levels among nurses who listened attentively to patients’ stories through “Zhizhi” morning meetings and narrative case presentations, their courage also increased significantly (*p* < 0.01). In the implementation of the humanistic care model, nurses have enhanced their understanding of humanistic care through comprehensive and systematic training. They practice according to a clearly structured care process, which equips them with greater courage and confidence in facing the high-pressure demands of nursing work. Consequently, their capacity for providing compassionate care has further improved. However, the research findings rely on self-assessment scales completed by nurses, lacking objective evaluations of humanistic care behaviors from patients or third-party observers. This may introduce social desirability bias. Future studies could incorporate multidimensional assessments, such as peer evaluations and patient satisfaction surveys, to enhance the reliability of the results.

The Medical narrative ability enables nurses to better attend to the inner world of patients, actively listening to their stories and emotions. This approach not only enhances patients’ emotional well-being but also encourages them to adopt a more positive attitude toward treatment and rehabilitation processes (37, 38) Furthermore, it fosters empathy among nurses, thereby improving their narrative communication skills and humanistic qualities (39). We implement a variety of approaches, including narrative training courses, exchanges of narrative nursing experiences and exemplary case sharing, thematic group discussions on narratives, and specialized narrative nursing rounds. These initiatives aim to comprehensively enhance the narrative literacy and knowledge of clinical nurses while further refining their narrative skills and empathetic techniques. The results indicate that the medical narrative ability improved from a pre-implementation score of (167.99 ± 21.67) to a post-implementation score of (177.29 ± 11.49) (*p* < 0.01). This demonstrates a significant enhancement in nurses’ medical narrative abilities following the intervention. Through narrative nursing rounds, role-playing, and situational simulations, we facilitate in-depth communication between nurses and patients. This approach allows nurses to listen attentively to patients’ stories and concerns, thereby stimulating their own emotional engagement. As a result, the ability of nurses to focus on active listening has significantly improved, with dimension scores showing a marked increase (*p* < 0.01). Through the summarization of experiences and the sharing of insights, we enhance our in-depth understanding of patients’ disease narratives. This practice effectively addresses the actual needs of patients, resulting in a significant improvement in our ability to comprehend and respond (*p* < 0.01). During the process of organizing case materials, the emotional fluctuations experienced in authentic scenarios allowed nurses to empathize with patients’ behaviors. This significantly enhanced their understanding and reflective capabilities regarding post-event analysis (*p* < 0.01). Clinical nurses, in their practice, focus on actively listening to patients’ narratives, understanding and responding to their needs. They analyze and interpret patients’ stories and confusions, gradually enhancing their reflective and representational skills. This process fosters empathy among nurses and significantly improves their medical narrative competence. The study did not conduct subgroup analyses regarding the nurses’ educational background, years of work experience, or prior narrative training experiences. For instance, more experienced nurses may master narrative techniques more quickly due to their extensive clinical experience, while less experienced nurses might require a longer adaptation period owing to insufficient communication experience. It is essential to explore the moderating effects of these background factors on the intervention outcomes.

The overall score and scoring rate for the practice of humanistic care among nurses in our country are relatively low (scoring rate < 80%) (40). The absence of humanistic care behaviors among nurses can lead to a decline in patient trust, medical experience, and overall satisfaction. This deficiency hinders the establishment and development of harmonious doctor-patient relationships (41) and serves as a potential catalyst for medical disputes (42). Research has confirmed that the nursing work environment is the primary factor influencing nurses’ humanistic care behaviors. This encompasses various aspects, including the physical environment, the cultural atmosphere of the workplace, and leadership dynamics (43). In this study, we established a “One Department, One Characteristic” humanistic care ward. We implemented convenient facilities to provide an optimal humanistic and physical environment. Additionally, we conducted activities aimed at cultivating humanistic care competencies, including Balint group counseling sessions, self-scheduling initiatives, Sleep Days, evaluations for head nurses, and reading corners. These efforts were designed to create a positive working atmosphere for nurses and address their needs for humanistic care. As a result of these interventions, the score for nurse caring behaviors increased from (131.65 ± 16.44) before implementation to (136.83 ± 8.69) after implementation (*p* < 0.01). Through humanistic training, nurses can gain a deeper understanding of the connotations, principles, and methods of caring behaviors. This training not only enhances their communication skills, empathy, and problem-solving abilities but also enables them to effectively apply humanistic care practices in their actual work. As a result, there is a significant improvement in nurses’ knowledge and skills (*p* < 0.01). The nurses consistently uphold a strong sense of responsibility for their own actions, demonstrating meticulous emotional care. They fully respect and attend to the needs of patients while actively expressing compassion and understanding toward them. The dimensions of respect and connection have significantly improved compared to before implementation (*p* < 0.01). In the realm of humanistic practice, it is essential to ensure that nurses receive adequate respect and care. Providing both material and emotional support enables nurses to experience the warmth and compassion of the hospital environment, thereby enhancing their confidence and hope in life. Furthermore, it is crucial to guarantee that nurses work in a fair and just setting where they can enjoy their rightful entitlements. This approach has been shown to significantly improve support and assurance dimensions (*p* < 0.05) compared to pre-implementation levels. Therefore, by deeply integrating humanistic care practices into clinical settings, nurses will be better equipped to fulfill their caregiving responsibilities, ultimately delivering higher quality and more compassionate nursing services for patients (44). The homogeneity and standardization of intervention measures are insufficient, which may lead to discrepancies in the implementation of humanistic ward creation across different departments. For instance, unquantified factors such as the leadership style of head nurses and team cohesion could interfere with the effectiveness of interventions. Therefore, it is essential to reduce bias through standardized operational procedures and process quality control.

### 5.3 Nurses perceive that the hospital attaches more importance to their care, and nurses’ satisfaction increases

As research on compassionate care continues to advance, the focus of compassion extends beyond nurses’ care for patients; it also encompasses the concern that nursing managers have for their staff (45). Research indicates that the care provided by nurse managers can significantly influence nurses’ loyalty to their profession and sense of team belonging. This support effectively reduces occupational stress and turnover rates among nurses, while simultaneously enhancing their work motivation and job satisfaction (46, 47). This study embodies the principle of prioritizing employees, ensuring that nurses receive a series of humanistic care initiatives based on the protection of their rights and interests. These measures aim to guarantee that nurses feel fully respected and supported in both their professional and personal lives, thereby effectively enhancing their job satisfaction and work motivation. The results indicate that nurses perceive an increased level of care and attention from the hospital compared to previous assessments (*p* < 0.01). Additionally, there is a significant improvement in nurse satisfaction (*p* < 0.001), suggesting that nurses feel valued and have a positive outlook on the current state of humanistic execution capabilities. Research indicates that the level of care experienced by nurses is positively correlated with their ability to provide care. Specifically, the more care nurses personally receive, the stronger their capacity to extend care to others becomes (48). This enhanced capability enables nurses to approach their work and patients with a more positive and constructive mindset, thereby indirectly improving the quality of nursing services and patient satisfaction. Nurse managers should focus on guiding nurses to develop a correct understanding of their work, addressing their reasonable needs, and fostering harmonious relationships. It is essential to create a positive working environment while enhancing humanistic care and supporting nurses’ career planning. This approach enables nurses to derive a sense of achievement from their work and boosts their confidence in professional development. The study was conducted in only one hospital, which limited the sample source and did not include nurses from different regions or various levels of healthcare institutions, thereby reducing the generalizability of the conclusions. Additionally, we measured only the short-term perceptions of care and satisfaction among nurses post-intervention, lacking long-term follow-up data to verify the sustainability of the intervention effects.

### 5.4 Effective humanistic care enhances patient satisfaction, and patients’ evaluations of nursing care behaviors are further improved

The patient satisfaction level serves as an indicator for evaluating the alignment between patients’ expectations of healthcare services and their actual perceptions of those services (49). This study adheres to the principle of “patient-centered,” creating an accessible inpatient environment. It refines service processes and fosters close collaboration between medical staff and nurses, implementing comprehensive, continuous, and seamless compassionate care. This approach encourages proactive communication and interaction between nurses and patients. The management emphasizes the cultivation of nurses’ awareness and skills in providing care, enabling patients to directly experience the concern and warmth of the nursing staff. Nurses ensure accurate information transmission by adhering to standardized communication techniques, such as clear, concise, and empathetic language. This approach fosters trust between nurses and patients, enhances the understanding of patients’ needs and feelings, and minimizes misunderstandings and conflicts. The evaluation of nurses’ caring behaviors and patient satisfaction among hospitalized patients has significantly increased compared to previous assessments (*p* < 0.001). This indicates that the implemented care measures are both feasible and effective, allowing patients to not only perceive the compassion, professionalism, and sense of responsibility exhibited by nurses but also fostering greater trust and recognition toward healthcare personnel. The study did not conduct stratified analyses based on factors such as age, disease type, and length of hospitalization. For instance, patients with chronic illnesses may prioritize the establishment of long-term relationships, whereas surgical patients might place greater emphasis on immediate communication, however, the research failed to differentiate these varying needs. The observed increase in satisfaction could be partially attributed to collaborative efforts among medical teams or advancements in medical technology rather than solely due to nursing care interventions. Nonetheless, the study lacked a control group designed to exclude confounding influences.

## 6 Conclusion

This study integrates the humanistic care nursing model into nursing practice, emphasizing participation, experience, and interaction. This approach effectively enhances nurses’ abilities in humanistic care, medical narrative skills, and caring behaviors. As a result, nurses have felt the love and concern from their managers. The model has received recognition from nurses, instilling them with greater confidence and courage to face high-pressure work environments. Concurrently, patients’ perception of nurses’ caring behaviors has significantly improved, leading to a notable increase in patient satisfaction. This finding is worthy of clinical promotion and application. However, this study does have limitations, such as being based on a small sample size and not exploring additional indicators. Future research will aim for targeted improvements to enhance the objectivity and scientific rigor of the findings.

## 7 Practical applications

Our research makes a significant contribution by building a humanistic care nursing model of staff “first” and “patient-centered,” placing both nurses and patients on an equally important pedestal. This model clearly defines practical humanistic care measures and behaviors. The implementation of the humanistic care model effectively enhances nurses’ capabilities in providing humanistic care, medical narrative skills, and caring behaviors. Consequently, patient satisfaction increases, leading to improved evaluations of nurses by patients.

Nursing managers should pay special attention to the psychological well-being and quality of life of nurses, while also providing adequate social support. They ought to enhance humanistic management practices, placing greater emphasis on people-centered approaches that address both the material and emotional needs of nurses. This support is essential for enabling nurses to realize their full professional potential and deliver higher-quality care services to patients.

## Data Availability

The raw data supporting the conclusions of this article will be made available by the authors, without undue reservation.
